# Evidence of an active Cushing reflex in a preterm neonate with hyaline membrane disease: a case report

**DOI:** 10.1186/s13256-021-03161-1

**Published:** 2021-12-14

**Authors:** Alan D. Rothberg, Johan Smith, Welma Lubbe

**Affiliations:** 1 School of Therapeutic Sciences, Faculty of Health Sciences, University of the Witwatersrand, Parktown, Johannesburg, 2193 South Africa; 2 Division of Neonatology, Department of Paediatrics and Child Health, Stellenbosch University, Stellenbosch, South Africa; 3 School of Nursing Science, North-West University, Potchefstroom, South Africa

**Keywords:** Cushing reflex, Tension pneumothorax, Intraventricular hemorrhage, Raised intracranial pressure, Background

## Abstract

**Background:**

The Cushing reflex does not appear to have been described in preterm neonates. This case report shows the presence of an active Cushing reflex in a 32-week preterm neonate with hyaline membrane disease.

**Case presentation:**

The 1.94 kg Caucasian infant was delivered by caesarean section following concerns about possible maternal infection and fetal compromise. Chest X-ray showed mild-to-moderate hyaline membrane disease and treatment was initiated with supplemental oxygen and nasal continuous positive airway pressure. It is probable that a pneumothorax occurred at 5–6 hours of age, with progression during the day. Interstitial air, pneumomediastinum, and tension pneumothorax were diagnosed on subsequent X-ray, and ultrasound of the brain showed a grade IV intraventricular hemorrhage. A review of the nurses’ recordings of heart rate, blood pressure, and respiratory rate showed a progressive increase in blood pressure accompanied by slowing of the heart rate and irregular respiration. These are features of the Cushing reflex that is elicited in response to raised intracranial pressure.

**Conclusion:**

While well-described in older children and adults, in neonates the Cushing reflex has mainly been described in animal experiments and infants who have developed hydrocephalus. It is likely that in this case, the reflex was elicited as a result of a progressive increase in intracranial pressure due to the combination of elevated intrathoracic pressure, obstructed venous return from the brain, and concurrent intraventricular hemorrhage.

The case presented shows the clinical course of a 32-week neonate with hyaline membrane disease (HMD) admitted in 2008 to a Level 2 Neonatal Intensive Care Unit (NICU) in a mid-sized South African city. In the absence of specialist neonatologists in that city, care was provided by a generalist pediatrician. In 2018, after 10 years of discussion between various parties, the case found its way into the courts and the authors of this article were engaged to provide expert opinion. From a clinical perspective, the uniqueness of this report is the evidence of an active Cushing reflex in a preterm neonate.

## Presentation of the case

The mother was admitted in preterm labor. Corticosteroids were immediately administered but a cesarean section was performed 10 hours later following concerns about a non-reassuring cardiotocographic tracing. The female infant, weighing 1.94 kg, was delivered with Apgar scores of 9 and 10 at 1 and 5 minutes, respectively. The attending pediatrician ordered immediate admission to the NICU, a unit staffed by nurses operating under the direction of pediatricians in private practice, not based in the hospital but available at any time if and when required.

**Fig. 1:**
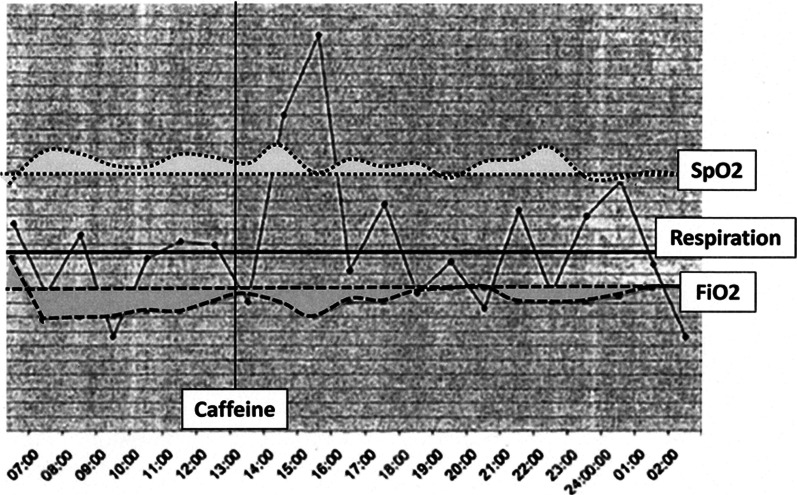
‘Continuous’ respiratory rate, inspired oxygen, and oxygen saturation traces derived from hourly recordings on nursing charts. SpO_2_ maintained between 87% and 95%; FiO_2_ maximum 0.45; dark line for respiratory rate = 60 breaths per minute

On admission, the infant was noted to be grunting with a respiratory rate of 72 breaths per minute. Nasal continuous positive airway pressure (NCPAP) was applied at 5 cm H_2_O, and inspired oxygen (FiO_2_) set at 0.6. Oximetry showed saturation (SpO_2_) of 95%. Venous blood gas was recorded as pH 7.24; pCO_2_ 56 mmHg; pO_2_ 72 mmHg; bicarbonate 21.9 meq/l. Chest X-ray was reported as showing mild-to-moderate HMD. Based on experience with previous infants of similar weight and gestational age, the attending pediatrician elected to continue with NCPAP, maintaining SpO_2_ between 87% and 95% with FiO_2_ ≤ 0.45. Routine admission blood tests including blood cultures were non-contributory. Routine observations included general assessment of neurological function and palpation of the anterior fontanelle. As shown in Fig. 1, for the ± 5 hours following admission, the routine hourly observations of respiratory parameters (rate, FiO_2_, SpO_2_) did not raise specific concerns. Occasional mild desaturations and brief periods of increasing oxygen requirements were noted, all of which responded to adjustments in FiO_2_. However increasing oxygen requirements from around 11:30 were reported to the attending doctor, who at 13:00 prescribed caffeine.

Shortly after the administration of caffeine there was a drop in respiratory rate, followed by improvements in SpO_2_ and the ability to again reduce FiO_2_. Nursing staff attributed the subsequent increases in respiratory rate to >100 breaths per minute to the caffeine. The pediatrician examined the infant again at around 15:00 and was satisfied with the condition. At no time were nurses alerted by monitoring device alarms to significant bradycardia, apnea, or hypotension, and based on the parameters shown in the figure, were not duly concerned about clinical deterioration until shortly before 02:50 when the infant collapsed. Resuscitation was initiated by the nursing staff, emergency department doctors were immediately summoned to assist, and the attending pediatrician was called to come in urgently. Resuscitation included intubation, initiation of mechanical ventilation, and administration of sodium bicarbonate, calcium, adrenaline, and midazolam. Chest X-ray at the time of collapse showed a left tension pneumothorax, pneumomediastinum, and significant pulmonary interstitial emphysema. An intercostal drain was inserted on the left. Subsequent ultrasound of the brain showed a grade IV intraventricular hemorrhage (IVH) [1]. The infant was discharged from hospital after 3 weeks but was subsequently diagnosed with cognitive impairment and cerebral palsy.

**Fig. 2:**
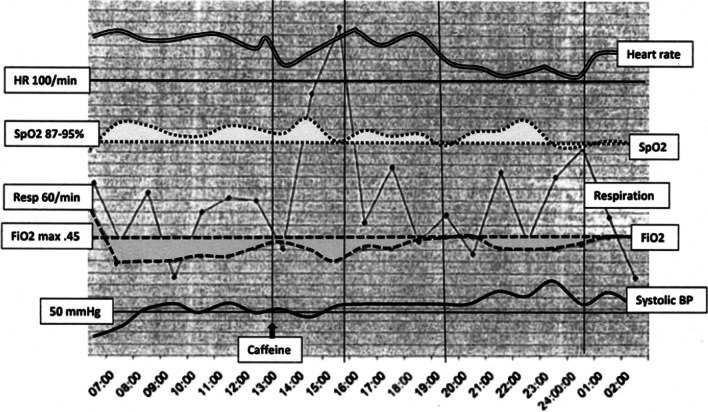
‘Continuous’ heart rate and blood pressure traces derived from hourly recordings on nursing charts and superimposed on the respiratory parameters in Fig. 1. Text boxes on left provide reference levels

Sick preterm neonates with significant and progressive disease typically present with evidence of falling blood pressure and episodes of bradycardia and apnea during deterioration. In this infant, regular observations of cardiorespiratory and general neurological status from the time of admission, as well as the infant’s responses to their interventions, therefore appeared to the nursing staff to be satisfactory prior to the collapse shortly before 03:00. However, when cardiovascular parameters of heart rate and blood pressure were superimposed on the respiratory traces, a very different picture emerged. Five discrete phases appeared to be identifiable (Fig. 2):

- Phase 1: 06:30–13:00; post-admission stabilization but increasing oxygen requirements from 11:30
- Phase 2: 13:00–16:00; a clinical event pre- or post-caffeine administration (most likely pneumothorax)
- Phase 3: 16:00–20:00; post-event stabilization
- Phase 4: 20:00–00:30; disturbances typical of Cushing reflex
- Phase 5: Decompensation and collapse.

At the onset of Phase 2, three aberrations occurred that are clearly visible on the composite graph: heart rate, respiratory rate, and blood pressure all decreased. These changes have been described and are consistent with the onset of pneumothorax [2]. However, even in the absence of intervention, the heart rate and blood pressure returned to previous levels, while respiratory rate increased dramatically. With the added benefit of hindsight (provided by the subsequent radiological confirmation of air leaks), the aberrations at the onset of Phase 2 were interpreted by the authors as most likely the result of acute pneumothorax. This was possibly precipitated by the caffeine, although the increasing oxygen requirements prior to the administration of caffeine might have been signs of a developing air leak and not simply progression of the HMD.

As shown in Fig. 2, the infant appears to have temporarily accommodated the pathology during Phase 3, but the subsequent changes in Phase 4 were dramatic. Heart rate slowed to low (∼100 beats per minutes), systolic blood pressure increased, and respiratory peaks became higher. These are features of the Cushing reflex, which is a response to raised intracranial pressure (ICP) and is characterized by the triad of slowing of the heart rate, elevations of blood pressure, and irregular respiration [3]. One can only speculate on what was transpiring intracranially between 19:30 and 00:30, but since tension pneumothorax and IVH were subsequently confirmed, it is reasonable to propose that both were present during Phase 4 and played a role in raising ICP, with the tension pneumothorax possibly predisposing to the IVH [4].

## Discussion

The outcome in this case would most likely have been different if surfactant had been administered. While not strictly in accordance with prevailing international guidelines recommending that surfactant should be administered to smaller preterm infants with HMD if FiO_2_ requirement exceeded 0.3 [5], faced with a 32-week neonate weighing 1.94 kg, the pediatrician was justified in initiating treatment with NCPAP and upper FiO_2_ limit of 0.45. This approach is supported by a Danish retrospective review of neonates born between 2000 and 2013 after 32–34 weeks gestation, treated with early NCPAP and FiO_2_ threshold of 0.4–0.5 [6]. In the absence of early surfactant administration, intervention in this case would have been appropriate towards the end of Phase 1, when the oxygen requirements were increasing. An X-ray might have shown a developing pneumothorax, while absence of such pathology would have been an indication to administer a ‘rescue’ dose of surfactant. Either or both of these interventions would likely have avoided the clinical situation that ultimately elicited the Cushing reflex.

Some discussion around the administration of caffeine and its possible role in pneumothorax is warranted. Caffeine is considered to be a safe drug and pneumothorax is not specifically recognized as a complication of its administration. However, caffeine is known to increase minute ventilation through increases in tidal volume and respiratory muscle activity [7]. In the presence of moderate and deteriorating HMD, where there is unequal expansion of alveoli and transpulmonary pressure is potentially being driven by caffeine, it is plausible that there may be rupture of overdistended alveoli, passage of air into perivascular sheaths, and development of pneumomediastinum and/or pneumothorax [8]. In the context of spontaneous pneumothorax in the term newborn, transpulmonary pressure may be as high as 120 cm H_2_O [8]. It is therefore conceivable that in a 1.94 kg surfactant-deficient neonate whose respiration is not only being driven by HMD but also by caffeine, transpulmonary pressure superimposed on a non-compliant and unevenly-aerated lung may have been sufficient to cause the air leaks that were subsequently seen on X-ray. A question arises in this case as to whether caffeine should have been administered at all. The answer is that it probably should not. As per Fig. 1, which shows the FiO_2_ rising to 0.45 after 5–6 hours, NCPAP was failing and treatment with surfactant should have been administered at that time. Also worth pointing out is that the elevated respiratory rates shown in Fig. 1 between 15:00 and 16:00 were not consistent with a caffeine effect. While the drug drives minute ventilation, it does so by increasing tidal volume and not by driving respiratory rate [7].

In patients older than the neonate under discussion, the Cushing’s triad of an increase in systolic blood pressure, slowing of the heart rate, and respiratory irregularity is recognized as an indication of raised ICP. Generally attributed to the body’s response to acutely elevated ICP within a rigid cranial vault and a sign of impending herniation, data are limited for neonates. The open sutures of the neonatal skull no doubt allow some accommodation if/when there is raised ICP, but there are likely limits to the accommodation and it is reasonable to assume that cerebral blood flow may be compromised if there is a significant acute to sub-acute increase in ICP. There is evidence that the reflex exists in neonatal rabbits, manifesting as hypertension, bradycardia, and decreased respiration in response to progressive increases in ICP [9]. The reflex has also been demonstrated in an 11-day old neonate with a rapidly expanding brain tumor [10] and in slightly older infants with hydrocephalus [11, 12]. In the infant under discussion, as shown in Fig. 2, there was a progressive slowing of the heart rate (albeit not to levels that nursing staff would classify as bradycardia) accompanied by progressively increasing blood pressure. During this period there were also wide swings in the respiratory rate, but without any episodes of apnea. We postulate that these changes reflect progressively increasing ICP in response to the accumulations of intrathoracic air and pressure, and the consequential impact on return of venous blood from the brain [4]. As elegantly shown by others, there is also congruence between development of pneumothorax and subsequent IVH [4]. Under these circumstances, a loss of cerebral vascular autoregulation may compound the problem [4]. Of particular interest during this infant’s deterioration is the SpO_2_, which, at around 22:30, reached the highest level of the day, while the FiO_2_ was actually being dialed down (Fig. 2). We propose that this was the result of greater peripheral perfusion in response to the elevated blood pressure. From a nursing perspective, these subtle manifestations of the Cushing reflex (the ‘settling’ of the heart rate and increases in blood pressure) were incorrectly read as reassuring signs, effectively masking the indicators of clinical deterioration. One can only speculate what transpired after midnight and ultimately led to the infant’s collapse, but based on the aforementioned animal experiments, it is likely that the ICP became critical and initiated coning [9].

## Conclusion

It is certainly possible that the Cushing reflex only manifested in this case because the opportunity was missed to treat the infant at ± 6 hours of age, thereby allowing a pathological situation to progress to the point of critically raised ICP. Nonetheless, it is important to note that there may be subtle manifestations of the reflex in a critically ill preterm infant such as this, even in the presence of a compliant cranium with open fontanelles and sutures.

## Data Availability

Patient data remain the property of the hospital in which the patient was treated and are not available for further scrutiny.

## Abbreviations

FiO_2_: Fractional inspired oxygen
HIE: Hypoxic ischemic encephalopathy
HMD: Hyaline membrane disease
ICP: Intracranial pressure
IVH: Intraventricular hemorrhage
NCPAP: Nasal continuous positive airway pressure
NICU: Neonatal intensive care unit
pO_2_: Partial pressure of oxygen
SpO_2_: Oxygen saturation of hemoglobin

## References

[1] Papile L, Burstein J, Burstein R. Incidence and evolution of subependymal and intraventricular hemorrhage: a study of infants with birth weights less than 1500 gm. J Pediatr. 1978;92:529–34. 10.1016/s0022-3476(78)80282-0 305471

[2] Ogata ES, Gregory GA, Kitterman JA, Phibbs RH, Tooley WH. Pneumothorax in the respiratory distress syndrome: incidence and effect on vital signs, blood gases and pH. Pediatrics. 1976;58:177–83. 7768

[3] Fodstad H, Kelly PJ, Buchfelder M. History of the Cushing reflex. Neurosurgery. 2006;59:1132–7. 10.1227/01.NEU.0000245582.08532.7C 17143247

[4] Hill A, Perlman JM, Volpe JJ. Relationship of pneumothorax to occurrence of intraventricular hemorrhage in the premature newborn. Pediatrics. 1982;69:144–9. 6799932

[5] Sweet D, Bevilacqua G, Carnielli V, Greisen G, Plavka R, Saugstad OD, et al. European consensus guidelines on the management of neonatal respiratory distress syndrome. J Perinat Med. 2007;35:175–86. 10.1515/JPM.2007.048 17480144

[6] Wiingreen R, Greisen G, Ebbesen F, et al. Surfactant need by gestation for very preterm babies initiated on early nasal CPAP: a Danish observational multicenter study of 6,628 infants born 2000–2013. Neonatology. 2017;111:331–6. 10.1159/000451021 28073106

[7] Dobson NR, Patel RM, Smith PB, Kuehn DR, Clark J, Vyas-Read S, et al. Trends in caffeine use and association between clinical outcomes and timing of therapy in very-low-birth-weight infants. J Pediatr. 2014;164:992–8. 10.1016/j.jpeds.2013.12.025 PMC3992195 24461786

[8] Ashmore PG. Spontaneous pneumothorax in the newborn. Can Med Assoc J. 1965;92:309–11. PMC1928107 14243865

[9] Ogilvy CS, DuBois AB. Effect of increased intracranial pressure on blood pressure, heart rate, respiration and catecholamine levels in neonatal and adult rabbits. Neonatology. 1987;52:327–36. 10.1159/000242728 3435736

[10] Wong CY, Azizi AB, Shareena A, Boo NY, Isa MR. Brain herniation in a neonate. Singap Med J. 2010;51:e166-168. 21103805

[11] Kalmar AF, Van Aken J, Caemaert J, Mortier JE, Struys MMRF. Value of Cushing reflex as warning sign for brain ischaemia during neuroendoscopy. Br J Anaesth. 2005;94:791–9. 10.1093/bja/aei121 15805143

[12] Uhrikova Z, Kolarovszki B, Javorka K, Javorka M, Matasova K, Kolarovszka H, Zibolen M. Changes in heart rate variability in a premature infant with hydrocephalus. AJP Rep. 2012;2:43–6. 10.1055/s-0032-1316462 PMC3653509 23946905

